# Effect of dietary supplementation with *Lactobacillus helveticus* R0052 on seizure thresholds and antiseizure potency of sodium valproate in mice

**DOI:** 10.1007/s00213-023-06489-2

**Published:** 2023-11-15

**Authors:** Piotr Wlaź, Adrian Wiater, Małgorzata Majewska, Elżbieta Wyska, Marcin Grąz, Joanna Śliwa-Dominiak, Nikola Gapińska, Katarzyna Socała

**Affiliations:** 1 Department of Animal Physiology and Pharmacology, Institute of Biological Sciences, Faculty of Biology and Biotechnology, Maria Curie-Skłodowska University, Akademicka 19, PL 20–033, Lublin, Poland; 2 Department of Industrial and Environmental Microbiology, Institute of Biological Sciences, Faculty of Biology and Biotechnology, Maria Curie-Skłodowska University, Akademicka 19, PL 20–033, Lublin, Poland; 3 Department of Pharmacokinetics and Physical Pharmacy, Jagiellonian University Medical College, Medyczna 9, PL 30–688, Kraków, Poland; 4 Department of Biochemistry and Biotechnology, Institute of Biological Sciences, Faculty of Biology and Biotechnology, Maria Curie-Skłodowska University, Akademicka 19, PL 20–033, Lublin, Poland; 5 R&D and Scientific Department, Sanprobi Sp. z o.o Sp.k., Quality Control and Microbiology Laboratory, Kurza Stopka 5/C, PL 70-535 Szczecin, Poland

**Keywords:** Gut-microbiota-brain axis, Psychobiotic, Excitation/inhibition balance, Anticonvulsant, Microbiota-derived metabolites

## Abstract

**Objective:**

Both animal and human studies, though limited, showed that multi-strain probiotic supplementation may reduce the number of seizures and/or seizure severity. Here, we evaluated the effect of a single strain probiotic supplementation on seizure susceptibility, antiseizure efficacy of sodium valproate, and several behavioral parameters in mice.

**Methods:**

*Lactobacillus helveticus* R0052 was given orally for 28 days. Its influence on seizure thresholds was evaluated in the *iv*PTZ- and electrically-induced seizure tests. The effect on the antiseizure potency of valproate was assessed in the *sc*PTZ test. We also investigated the effects of probiotic supplementation on anxiety-related behavior (in the elevated plus maze and light/dark box tests), motor coordination (in the accelerating rotarod test), neuromuscular strength (in the grip-strength test), and spontaneous locomotor activity. Serum and brain concentrations of valproate as well as cecal contents of SCFAs and lactate were determined using HPLC method.

**Results:**

*L. helveticus* R0052 significantly increased the threshold for the 6 Hz-induced psychomotor seizure. There was also a slight increase in the threshold for myoclonic and clonic seizure in the *iv*PTZ test. *L.* *helveticus* R0052 did not affect the threshold for tonic seizures both in the maximal electroshock- and *iv*PTZ-induced seizure tests. No changes in the antiseizure potency of valproate against the PTZ-induced seizures were reported. Interestingly, *L.* *helveticus* R0052 increased valproate concentration in serum, but not in the brain. Moreover, *L.* *helveticus* R0052 did not produce any significant effects on anxiety-related behavior, motor coordination, neuromuscular strength, and locomotor activity. *L.* *helveticus* R0052 supplementation resulted in increased concentrations of total SCFAs, acetate, and butyrate.

**Conclusions:**

Altogether, this study shows that a single-strain probiotic – *L.* *helveticus* R0052 may decrease seizure susceptibility and this effect can be mediated, at least in part, by increased production of SCFAs. In addition, *L.* *helveticus* R0052 may affect bioavailability of valproate, which warrants further investigations.

## Introduction

Epilepsy is a chronic disorder of the central nervous system (CNS) characterized by recurrent unprovoked seizures that develop as a result of excessive electrical discharges in the brain. Seizures are considered to be a consequence of imbalance between excitation and inhibition in the CNS caused by changes in GABAergic (inhibitory) and glutamatergic (excitatory) neurotransmission, disturbances in the function of ion channels and pumps, alterations in cell metabolism or energy and other factors. However, the exact mechanisms underlying epileptic seizures are still unclear (Jefferys 2010; Stafstrom and Carmant 2015). The etiology of epilepsy is complex, involving structural, metabolic, infectious, immune, genetic, and unknown causes. Also, the mechanism of epileptogenesis is multifactorial and poorly understood (Vezzani et al. 2016). Although pharmacotherapy remains the most common treatment option for epilepsy, in over 30% of patients seizures cannot be satisfactorily controlled with antiseizure medications (De Caro et al. 2019a). Furthermore, currently available antiseizure drugs possess only symptomatic activity – they do not prevent epileptogenesis and are devoid of disease-modifying properties (Galanopoulou et al. 2021). Thus, further studies are required to better understand the pathophysiology of epilepsy and to develop novel therapeutic strategies for epilepsy.

Accumulating evidence indicates the potential role of the gut microbiota in epilepsy. Several studies demonstrated the differences in the gut microbiota composition between patients suffering from epilepsy and healthy subjects. Differences were also reported between drug-resistant and drug-sensitive epileptic patients, and in patients treated with the ketogenic diet (Fusco et al. 2022; Kundu et al. 2023; Lum et al. 2020). Moreover, gut dysbiosis may be a common pathological factor between epilepsy and its comorbid disorders (Arulsamy and Shaikh 2022). Accordingly, remodeling gut microbiota with probiotics, prebiotics, synbiotics, personalized diet, or fecal microbiota transplantation has emerged as a possible therapeutic strategy in epilepsy (Iannone et al. 2020, 2022; Kundu et al. 2023).

Probiotics are defined as live microorganisms that alter intestinal microbiota and confer a health benefit on the host. Two human studies showed potential beneficial effects of probiotics in seizure/epilepsy management. In one study, a 4-month probiotic supplementation reduced seizure number and improved the Quality of Life score in patients with drug-resistant epilepsy (Gómez-Eguílaz et al. 2018). The second study showed that administration of probiotics after birth may decrease rotavirus-associated seizures in neonates (Yeom et al. 2019). In animal studies, probiotics treatment reduced acute penicillin- (Kızılaslan et al. 2022) and PTZ-induced seizures (Aygun et al. 2022a; Kilinc et al. 2021), suppressed development of PTZ-induced kindling (Bagheri et al. 2019; Tahmasebi et al. 2020), ameliorated spontaneous seizures after kainic acid-induced status epilepticus (Wang et al. 2022), and alleviated absence seizures in WAG/Rij rats with absence epilepsy (Aygun et al. 2022b).

*Lactobacillus helveticus* R0052 is a widely used probiotic strain originally isolated in 1990 from a North American dairy starter culture (Naser et al. 2006). Its beneficial effects have been proven in numerous in vitro and in vivo studies (Foster et al. 2011). For example, it was shown to elicit anti-inflammatory responses, affect the anxiety-related behavior in mice (Ohland et al. 2013), and alleviate liver injury in rats (Wang et al. 2019b). Moreover, combination of *L.* *helveticus* R0052 and *Bifidobacterium longum* R0175 is a well-known commercially available psychobiotic formulation (Kazemi et al. 2019; Messaoudi et al. 2011). The possible ability of *L.* *helveticus* R0052 to modulate the brain-gut axis suggests that it may also affect seizure susceptibility. Therefore, the aim of the present study was to evaluate the effect of a 28-day supplementation with *L.* *helveticus* R0052 on seizure thresholds in three acute seizure tests in mice. In addition, the effects on anxiety-like behavior, spontaneous locomotor activity, motor coordination, neuromuscular strength, and cecal levels of short chain fatty acids (SCFAs) and lactate were investigated. We also sought to assess the influence of *L.* *helveticus* R0052 on the antiseizure activity of sodium valproate. Valproate is a broad spectrum antiseizure drug commonly used for the treatment of generalized and unclassifiable epilepsy (Marson et al. 2021). According to the biopharmaceutics drug disposition classification system (BDDCS) classifications of oral drugs, valproate belongs to class 1 drugs that are characterized by high metabolism and high solubility. For class 1 drugs, metabolizing enzymes in the intestine play an important role. The gut microbial enzyme activity can potentially influence their pharmacokinetics and as a consequence therapeutic efficacy (Zhang et al. 2021). To evaluate whether manipulation of the gut microbiota by probiotic supplementation could affect valproate bioavailability, we determined serum and brain concentrations of valproate after *L.* *helveticus* R0052 intake.

## Materials and methods

### Animals

All experiments were carried out on male albino Swiss mice (n = 210). The animals were purchased from a licensed breeder (Kołacz, Laboratory Animals Breeding, Warsaw, Poland) at age of 5–6 weeks and kept under controlled environmental conditions (21–24 °C; 45–65% humidity; 12-h light/dark cycle; light on at 6:00 a.m.) in the conventional animal facility. Standard laboratory chow (Agropol S.J., Motycz, Poland) and tap water were available ad libitum. Food pellets and water were not sterilized. The bottles were washed, disinfected, and filled with fresh filtered water three times a week. Mice were housed in groups of 8 in standard transparent cages (37 cm × 21 cm × 14 cm) and habituated for 8 days before being used in the experiment. The experiments were performed during the light phase. The light/dark box test and the elevated plus maze test were conducted in a darkened room, while all other tests in a lighted experimental room.

Housing and experimental procedures were performed in accordance with the EU council directive 2010/63/EU and Polish legislation concerning animal experimentation. The euthanasia was performed by exposure to a gradually increasing concentration of carbon dioxide or by decapitation for blood and brain collection. All experimental protocols were approved by the Local Ethics Committee in Lublin, Poland (license no 68/2021).

### Preparation of bacterial strain

*Lactobacillus helveticus* R0052 was obtained courtesy of Sanprobi Sp. z o.o. Sp.k. (Szczecin, Poland). The bacterial suspension was prepared according to Kochalska et al. (2020), with some modifications. Briefly, bacterial strain was stored as a frozen stock at −80 °C in Man–Rogosa–Sharpe liquid medium (MRS broth; Difco Laboratories, Detroit, MI, USA) containing 20% glycerol. From frozen stocks, bacteria were sub-cultured (overnight, in anaerobic conditions, 37 °C) in the MRS medium supplemented with 0.05% L-cysteine-HCl. The overnight culture was transferred to fresh MRS broth (4.5 l) and again incubated at 37 °C for 48 h in anaerobic conditions (under mineral oil). After 2 days, cells were harvested from the growth medium by centrifugation at 8,150 × *g* for 20 min. The pellets were washed three times with sterile PBS and re-suspended in 450 ml of sterile phosphate-buffered saline (PBS). The turbidity of bacterial suspension was compared to the McFarland scale and its dilution was made in sterile PBS to 1 × 10^10^ cfu/ml (2 × 10^9^ cfu/0.2 ml). The final bacterial suspensions were bottled into 50-ml Falcon tubes and stored at −20 °C until the mice were fed.

### Study design and treatment

**Fig. 1:**
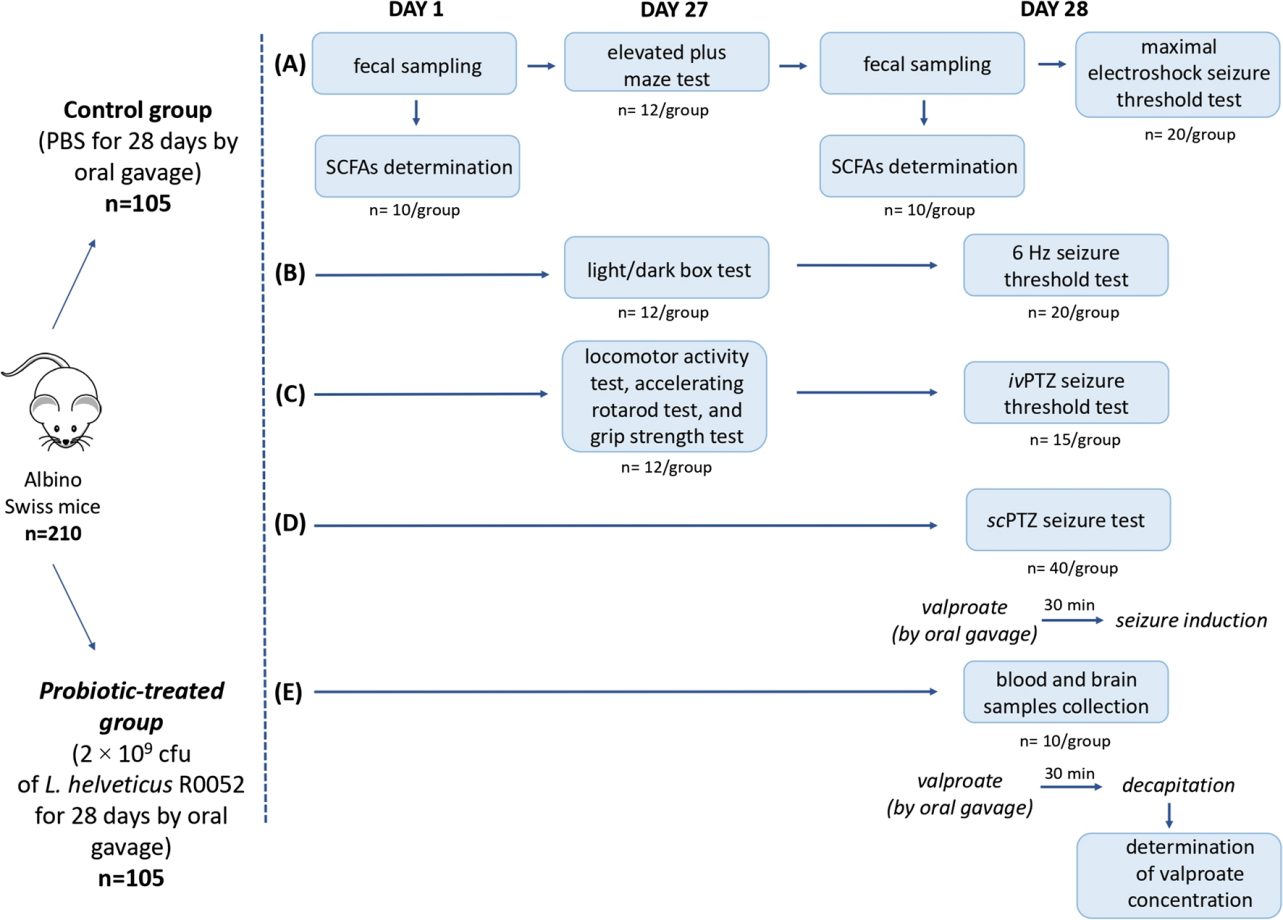
Schematic illustration of the experimental design

Mice were administered 2 × 10^9^ cfu of *L.* *helveticus* R0052 in 200 μl of PBS by oral gavage every 24 h for 28 days. Control animals received equivalent volume of PBS. Control and *L.* *helveticus* R0052-treated groups consisted of 105 mice each. All seizure tests were performed on day 28, 120 min after the last treatment. Behavioral tests (i.e., the elevated plus maze test, the light/dark box test, the locomotor activity test, the rotarod test and the grip strength test) were performed 24 h earlier (on day 27) to limit the number of animals used in the experiment. For this reason, the animals were divided into the following subgroups: (a) elevated plus maze test (12 mice/group) on day 27 and the maximal electroshock seizure threshold test (20 mice/group) on day 28; (b) light/dark box test (12 mice/group) on day 27 and the 6 Hz seizure threshold test (20 mice/group) on day 28; (c) locomotor activity test, accelerating rotarod test, and the grip strength test (12 mice/group) on day 27 and the *iv*PTZ test (15 mice/group) on day 28; (d) *sc*PTZ test (40 mice/group) on day 28; and (e) decapitation for determination of valproate concentration (10 mice/group) on day 28. Fecal samples for SCFAs analysis were collected from mice assigned to the first subgroup. The schematic representation of study design is shown in the Fig. 1.

### Maximal electroshock seizure threshold test

Electroconvulsions were induced by applying a sinusoidal alternating current (50 Hz; maximum output voltage 500 V, stimulus duration 200 ms) via transcorneal electrodes with the usage of rodent shocker (type 221; Hugo Sachs Elektronik, Freiburg, Germany). The seizure threshold was determined by the “up and down” method (Kimball et al. 1957) and expressed as the median convulsive current (CC_50_ in mA with confidence limits for 95% probability) predicted to produce the tonic hindlimb extension in 50% of the animals tested. All experimental details were described elsewhere (Socała et al. 2016; Socała and Wlaź 2021).

### Six hertz (6 Hz) psychomotor seizure threshold test

Psychomotor seizures were induced by corneal stimulation (0.2 ms rectangular pulse width at 6 Hz frequency, stimulus duration 3 s) with the usage of Grass S48 stimulator and CCU1 constant current unit (Grass Technologies, West Warwick, RI, USA). As in the maximal electroshock seizure threshold test, the 6 Hz seizure threshold test was determined by the “up and down” method (Kimball et al. 1957) and expressed as the CC_50_ values (with confidence limits for 95% probability), i.e., as the median convulsive current (in mA) needed to produce psychomotor seizures in 50% of the animals tested. The experimental procedure is described in detail in our earlier studies (Socała et al. 2016).

### Intravenous (*iv*) PTZ Seizure Threshold Test

A 1% solution of PTZ (Sigma Aldrich, St. Louis, MO, USA) was infused at a constant rate of 0.2 ml/min in freely moving mice. The time latencies from to the appearance of the first myoclonic twitch, generalized clonus with loss of righting reflex, and tonic forelimb extension were recorded. The seizure thresholds were expressed as the amount of PTZ (in mg/kg) needed to produce the first apparent sign of each endpoint. The method is described in detail elsewhere (Socała et al. 2016; Socała and Wlaź 2021).

### Subcutaneous (*sc*) PTZ seizure test

The *sc*PTZ seizure test was used to evaluate the effect of treatment with of *L.* *helveticus* R0052 on the antiseizure activity of valproate in mice. Sodium valproate (TCI, Tokyo, Japan) was dissolved in normal saline and administered by oral gavage at a constant volume of 10 ml/kg. Clonic seizures were produced by *sc* injection of PTZ at a dose of 96 mg/kg, equal to its predetermined CD_97_ value (i.e., the dose that induced clonic seizures in 97% of animals tested). Both control and probiotic-treated animals were subdivided into 5 subgroups (8 animals each) and administered with increasing doses of sodium valproate (200–500 mg/kg) 30 min before PTZ injection. Immediately following PTZ administration, the animals were placed separately into transparent cages and observed for the next 30 min for the presence or absence of clonic seizures. The number of animals protected from clonic seizures (out of the total number of mice) was noted for each subgroup. A log-probit method (Litchfield and Wilcoxon 1949) was used to determine the median effective doses (ED_50_) of valproate, i.e., doses (in mg/kg) that protected 50% of animals against the PTZ-induced clonic seizures.

### Elevated plus-maze test

The elevated plus-maze test was carried out according to the method described in detail previously (Socała and Wlaź 2016). Animals were automatically tracked during a 5-min trail using ANYmaze Video Tracking System (version 4.82, Stoelting Co., Wood Dale, IL, USA). Data were expressed as the percentage of open arms entries and the percentage of time spent in open arms.

### The light/dark box test

The light/dark transition test was performed as described previously (Socała and Wlaź 2016). Mice were allowed to explore the apparatus for 10 min. The trail was recorded and the number of light compartment entries, the time spent in the light compartment, and the latency to enter the dark compartment was scored by a trained observer.

### Locomotor activity test

Spontaneous locomotor activity was monitored for 15 min using an automated infrared beam-based IR Actimeter system supported by SedaCom32 software (Panlab/Harvard Apparatus, Barcelona, Spain) according to the method described in detail elsewhere (Socała and Wlaź 2016). After 5 min of acclimatization, the horizontal locomotor activity was monitored and expressed as a total number of beam breaks during a 10-min trial.

### Grip strength test

Neuromuscular strength in mice was measured using the grip-strength apparatus (BioSeb, Chaville, France) as described previously (Socała et al. 2016). The grip-strength was normalized to body weight and expressed in mN/g.

### Accelerating rotarod test

Animals were trained on the rotarod apparatus (Rotamex-5, Columbus Instruments, Columbus, OH, USA) for 3 consecutive days, 3 times per day with 120 min intervals between the training sessions. During each training session, mice were placed for 3 min on the rotating rod (4–24 rpm, constant speed) with unlimited trials. On the testing day, mice were placed on the rod accelerating from 0 to 40 rpm over 4 min and then maintained a constant speed for 1 min. The latency to fall and the rpm of the rod at the fall during three consecutive trials were automatically recorded and the data were averaged.

### Determination of valproate concentration in serum and brain

In order to determine the influence of treatment with *L.* *helveticus* R0052 on serum and brain valproate concentrations, both control and *L.* *helveticus* R0052-treated mice were administered by oral gavage with valproate at a dose of 380 mg/kg (close to its ED_50_ value from the *sc*PTZ test) and decapitated 30 min later. The trunk blood was collected into Eppendorf tubes, allowed to clot at room temperature, and then centrifuged at 3,000 × *g* for 10 min. The serum was transferred into fresh tubes at and stored at − 20 °C. Brains were dissected from the skull immediately after the decapitation, washed with 0.9% NaCl, and frozen at –20 °C until analysis.

Before extraction procedure, murine brains were homogenized in distilled water (1:4, w/v) using a TH220 tissue homogenizer (Omni International, Inc., Warrenton, VA, USA). To determine valproic acid concentrations in biological samples the method described by Zhang et al. (2014) was used with slight modifications. Briefly, to tubes containing 50 μl of serum or 250 μl of brain homogenate 10 μl of ibuprofen (internal standard) solution in methanol was added at concentrations of 2 mg/ml and 100 µg/ml, respectively. Then, the samples were acidified with 100 µl of 1 M sulfuric acid. After vortex-mixing for 1 min, the samples were extracted with 1 ml of hexane using a mechanical shaker for 15 min. After centrifugation at 10,000 × *g* for 5 min, 0.7 ml of organic layers were transferred to new tubes and 20 μl of potassium carbonate (0.01 M) was added. The mixture was evaporated to dryness under a gentle stream of nitrogen at 37 °C after vortex-mixing for 30 s. The residue was dissolved with 50 μl of 2-bromo-2′-acetonaphthone (10 mg/ml) and 10 μl of 18-crown-6 ether (1 mg/ml) both prepared in acetonitrile. The mixture was vortexed for 1 min and incubated in a dry heat sterilizer (model MOV-112S, Sanyo, Japan) at 65 °C for 25 min. The serum samples were diluted 10 times with acetonitrile before analysis. Subsequently, 5 μl aliquots of these mixtures were subjected to HPLC analysis.

The HPLC system consisted of an isocratic pump (model L-7100), an autosampler (model L-7200) both from Merck Hitachi (Darmstadt, Germany), and a UV variable-wavelength K-2600 detector (Knauer, Berlin, Germany) set at 251 nm. Data acquisition and processing were performed using the D-7000 HSM software (Merck Hitachi). The analysis was performed on a 250 × 4 mm LiChrospher1100 RP-18 column with a particle size of 5 mm (Merck, Darmstadt, Germany) protected with a guard column (4 × 4 mm) with the same packing material. Chromatographic analyses were carried out at room temperature. The mobile phase was composed of methanol and water mixed at a ratio of 80:20 (v/v). The calibration curves constructed by plotting the ratios of the peak area of valproic acid to the internal standard vs. corresponding valproic acid concentrations were linear in the tested concentration ranges (2–600 μg/ml(g), r > 0.99). No interfering peaks with the same retention times as the studied drug and the internal standard were observed in the chromatograms. The intra- and inter-assay precision was below 10%, whereas the intra- and inter-assay accuracy ranged from 94.7 to 105% of the theoretical target concentrations. The retention times were 15 min for the studied drug and 23 min for the internal standard. Valproic acid concentrations were expressed in μg/ml of serum and μg/g of brain tissue.

### Determination of SCFAs and lactate in fecal samples

Fecal samples were collected before and after probiotic/PBS treatment. Fecal pellets (2–3/mouse) were obtained by restraining the mouse and letting it defecate directly into sterile 1.5 ml tubes. Mice which did not defecate into the tube, were placed into an empty cage (with no bedding) and allowed to defecate normally. Immediately after defecation, the fecal pellets were transferred into sterile tubes and stored at −80 °C until analysis. Immediately after thawing, fecal samples (100 mg) were suspended in 1 ml of PBS and vortexed until a homogeneous suspension was obtained (about 20 min). The suspension was centrifuged at 14,500 × *g* for 10 min at 4 °C to remove the solid material, and the supernatants were collected and filtered through a membrane filter (pore size 0.22 μm). The quantification of lactate and SCFAs (acetate, propionate, butyrate, isobutyrate) in samples was performed according to Wang et al. (2019a) with some modifications. The HPLC system (Agilent Infinity 1260 equipped with DAD detector) fitted with a Bio-Rad Aminex HPX-87H (300 × 7.8 mm) column was operated at 30 °C with 0.008 N H_2_SO_4_ as the mobile phase at the flow rate of 0.55 ml/min and injection time set to 20 s. Peak identification was done by comparing with commercially available acetic, propionic, butyric, isobutyric and lactic acids (Sigma-Aldrich, St. Louis, MO, USA).

### Statistical analysis

Based on the data obtained in the maximal electroshock- and 6 Hz-induced seizure tests, the mean values of log mA (with standard deviations) were calculated according to the method described by Kimball et al. (1957) and analyzed using the unpaired Student’s t test. The data were then converted into CC_50_ values with confidence limits for 95% probability. Wilcoxon matched-pairs signed rank test was used to compare SCFAs and lactate concentrations in the same mice before and after treatment. The differences in ED_50_ values of valproate were assessed by Student’s t test. All remaining results were analyzed using non-parametric Mann–Whitney test. The normality of the data was assessed using D'Agostino & Pearson test.

Results were considered statistically significant when p value was less than 0.05. All the calculations were performed using GraphPad Prism 8.4.3 for Windows (GraphPad Software, San Diego, CA, USA).

## Results

### Effect of *L. helveticus* R0052 administration on seizure thresholds in the maximal electroshock- and 6 Hz-induced seizure tests

**Fig. 2:**
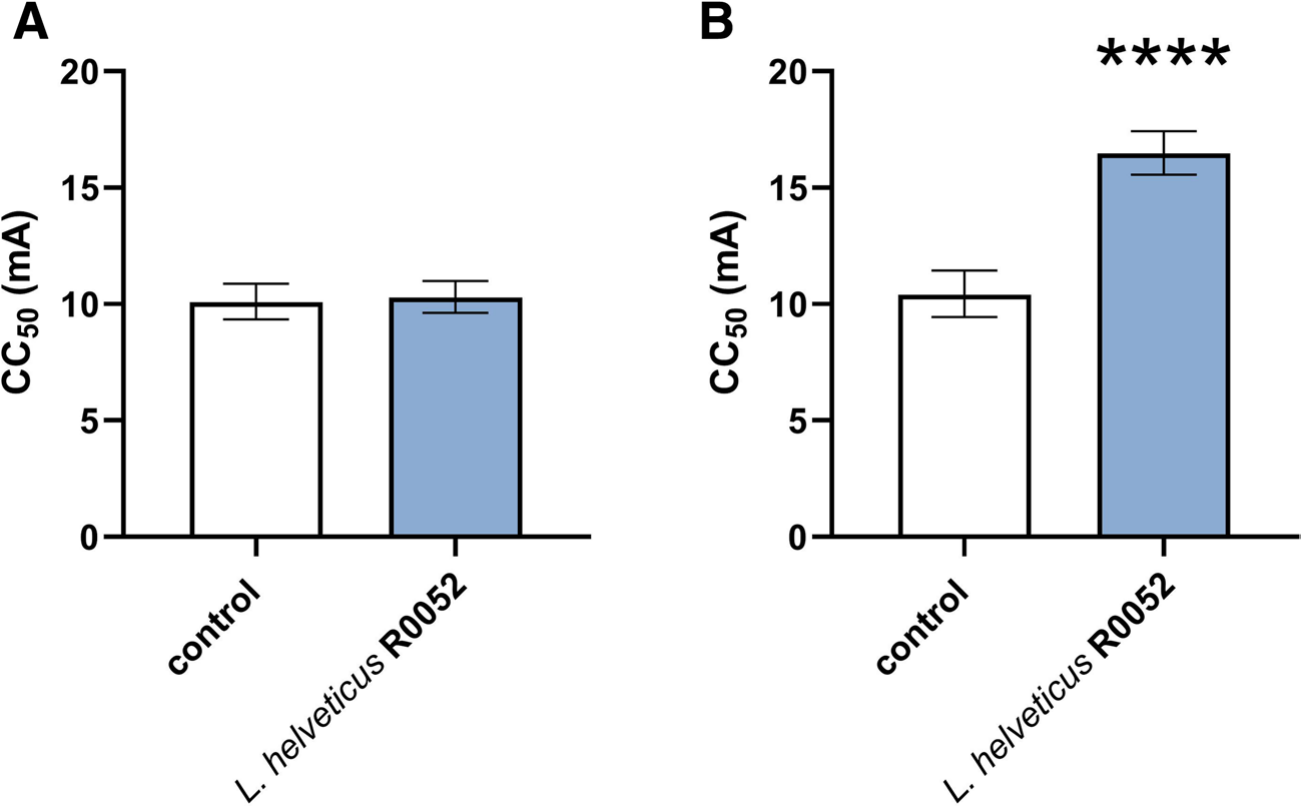
Effect of *L.* *helveticus* R0052 administration on the seizure threshold in the maximal electroshock seizure test (A) and the 6 Hz-induced seizure test (B) in mice. Mice were administered orally with 2 × 10^9^ cfu of *L.* *helveticus* R0052 for 28 days. Control animals received 200 μl of PBS. Seizure test was performed 120 min after last administration. Data are presented as CC_50_ values (in mA) with upper and lower 95% confidence limits. Each experimental group consisted of 20 animals. The statistical significance was evaluated using Student’s t test. ****p < 0.0001 vs. control group

As shown in Fig. 2A, a 28-day treatment with *L.* *helveticus* R0052 had no impact on the threshold for the tonic hindlimb extension in the maximal electroshock-induced seizure test in mice (t = 0.395, df = 17, p = 0.698). However, administration of *L.* *helveticus* R0052 caused a significant increase of the threshold for psychomotor seizures in the 6 Hz test. It raised the CC_50_ value by ~ 60% (t = 8.225, df = 16, p < 0.0001; Fig. 2B).

### Effect of *L.* *helveticus* R0052 administration on seizure thresholds in the timed ivPTZ seizure test

**Fig. 3:**
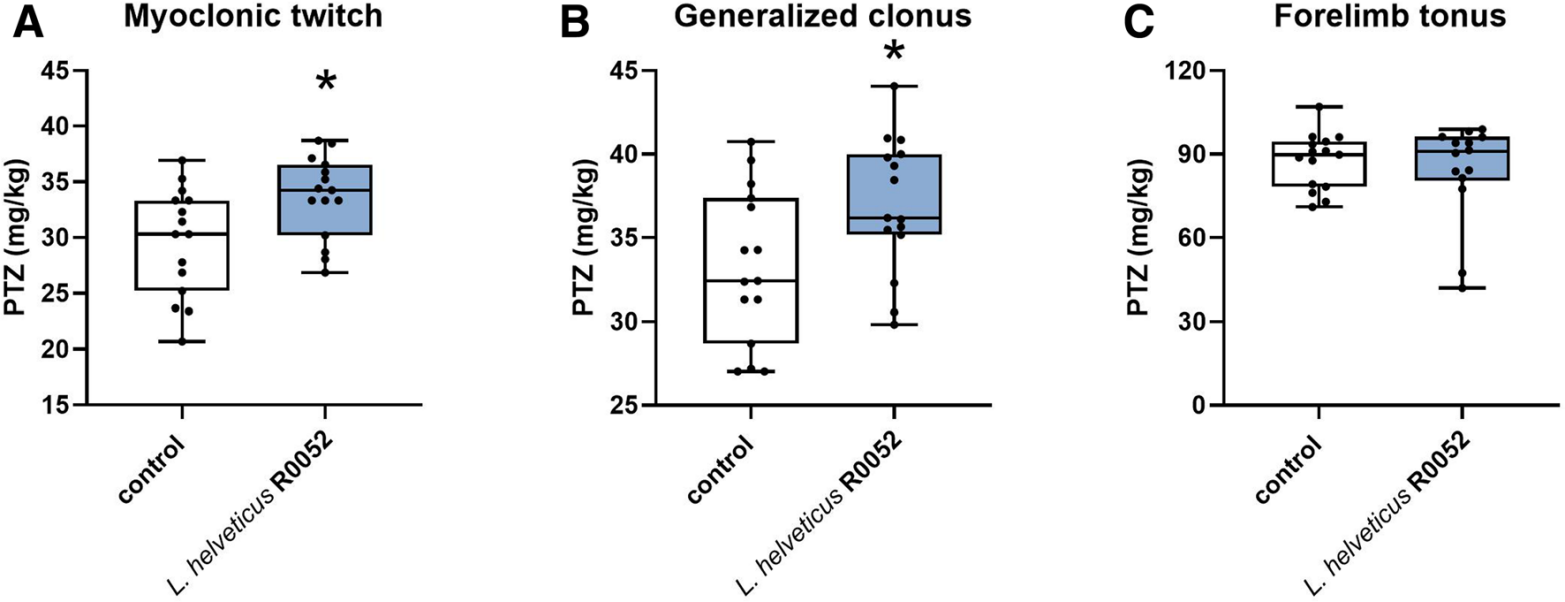
Effect of *L.* *helveticus* R0052 administration on the threshold for the first myoclonic twitch (A), generalized clonus (B), and forelimb tonus (C) in the *iv*PTZ seizure threshold test in mice. Mice were administered orally with 2 × 10^9^ cfu of *L.* *helveticus* R0052 for 28 days. Control animals received 200 μl of PBS. Seizure test was performed 120 min after last administration. Data are presented as box plots showing: interquartile range (the width of the box), minimum and maximum values (whiskers), and median (horizontal line). Each experimental group consisted of 15 animals. The statistical significance was evaluated using Mann–Whitney test. *p < 0.05 vs. control group

Repeated administration of *L.* *helveticus* R0052 produced a slight (~ 13%) but statistically significant increase of the threshold for the first myoclonic twitch (U = 57.5, p = 0.021). Likewise, *L.* *helveticus* R0052 very slightly (by ~ 11%) raised the threshold for the onset of generalized clonic seizures with loss of righting reflex (U = 62, p = 0.036). No statistically significant changes in the threshold for the forelimb tonic extension were observed (U = 98, p = 0.772). Data from the *iv*PTZ test are presented in Fig. 3A–C.

### Effect of *L.* *helveticus* R0052 administration on the anticonvulsant activity of valproate in the *sc*PTZ seizure test and valproate concentrations

**Fig. 4:**
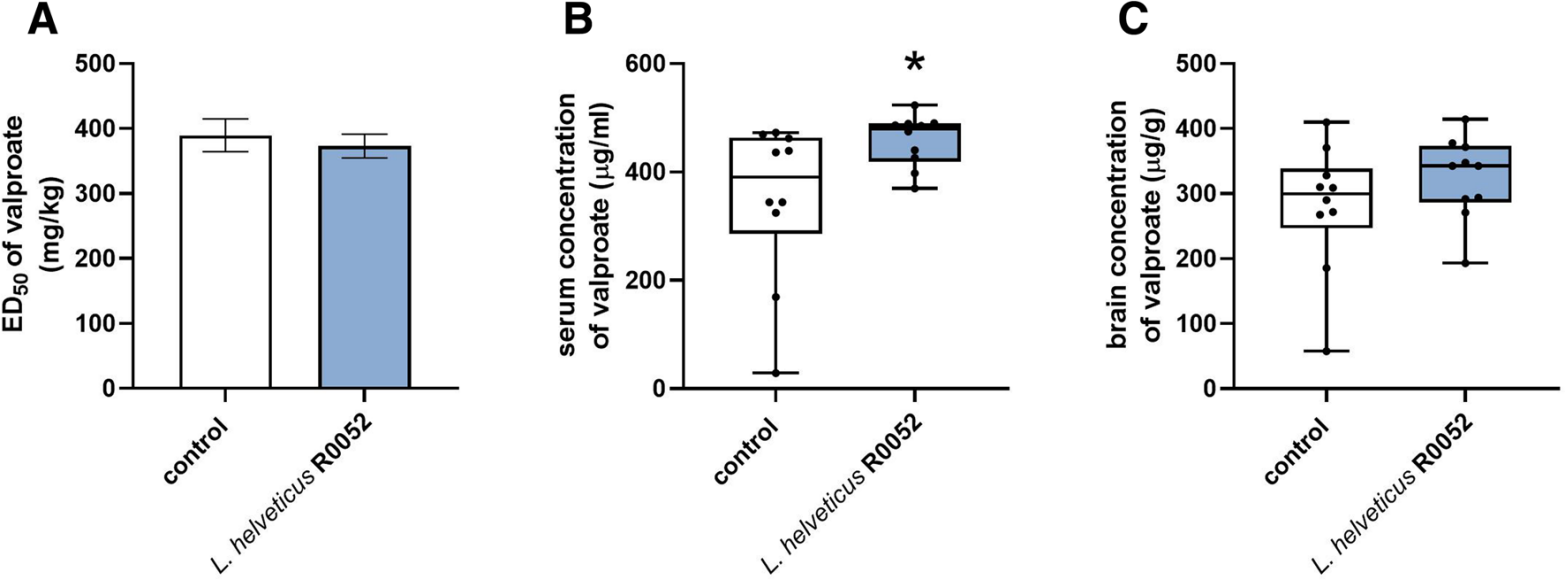
Effect of *L.* *helveticus* R0052 administration on the anticonvulsant potency of valproate in the *sc*PTZ test (A), and concentrations of valproate in serum (B) and brain (C) Mice were administered orally with 2 × 10^9^ cfu of *L.* *helveticus* R0052 (for 28 days. Control animals received 200 μl of PBS. Seizure test or decapitation was performed 120 min after last administration. Valproate was administered orally, 30 before PTZ injection or decapitation. ED_50_ (± SEM) values (determined in groups of 40 mice each) represent a dose of valproate predicted to protect 50% of mice tested against the *sc*PTZ-induced seizure. Valproate concentrations (determined in groups of 10 mice each) are presented as box plots showing: interquartile range (the width of the box), minimum and maximum values (whiskers), and median (horizontal line). The statistical significance was evaluated using Student’s t test (A) or Mann–Whitney test (B and C). *p < 0.05 vs. control group

A 28-day treatment with *L.* *helveticus* R0052 had no significant effect on the anticonvulsant potency of valproate against the *sc*PTZ-induced clonic seizures; no changes in ED_50_ values were reported (t = 0.479, df = 38, p = 0.635; Fig. 4A). However, *L.* *helveticus* R0052 administration caused a significant increase (by ~ 30%) of valproate concentration is serum (U = 18, p = 0.015; Fig. 4B). There were no changes in valproate concentration in the brain (U = 33, p = 0.218; Fig. 4C).

### Effect of *L.* *helveticus* R0052 administration on anxiety-like behavior in the elevated plus maze test and the light/dark box test

**Fig. 5:**
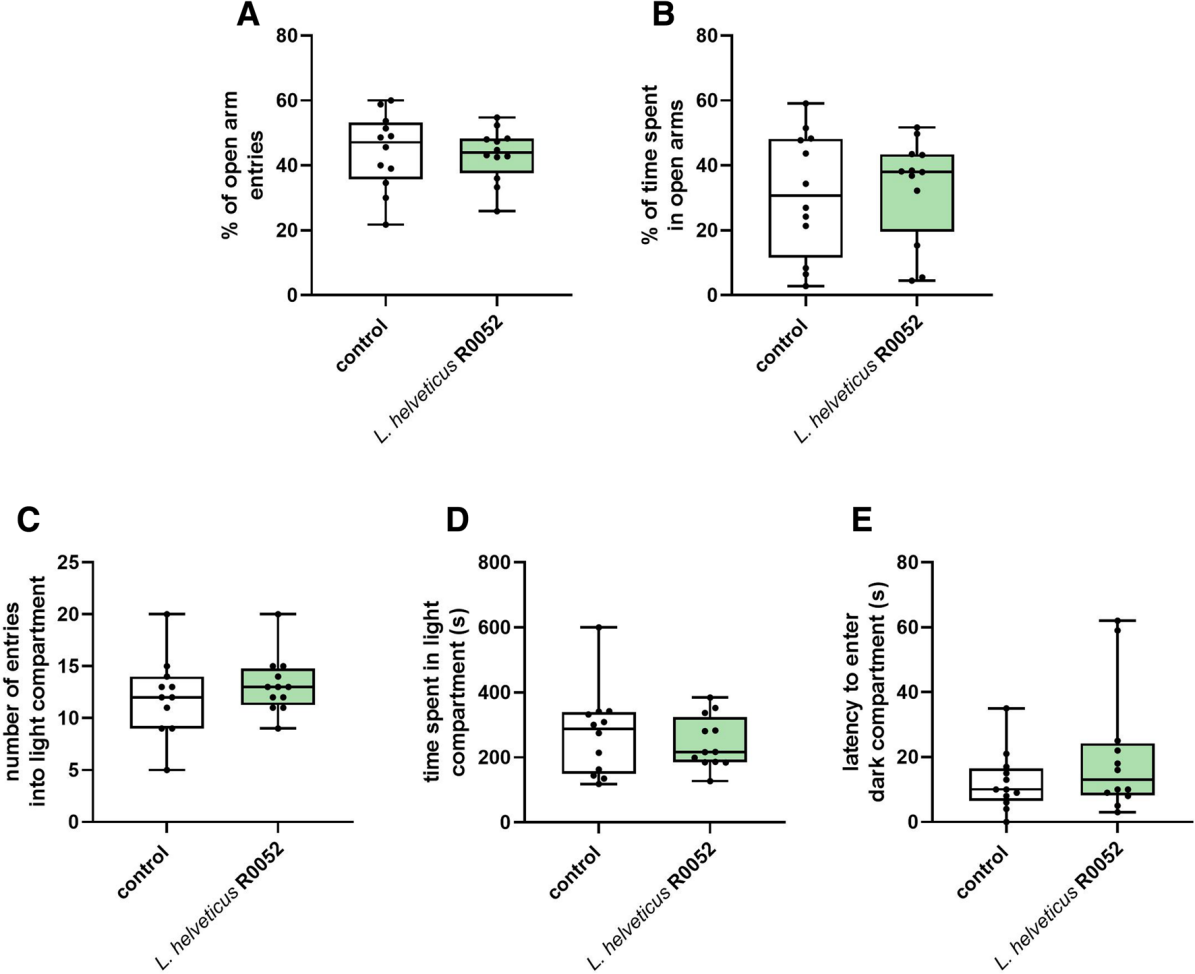
Effect of *L.* *helveticus* R0052 administration on anxiety-like behavior in mice assessed in the elevated plus maze test (A–B) and the light/dark box test (C–D). Mice were administered orally with 2 × 10^9^ cfu of *L.* *helveticus* R0052 for 27 days. Control animals received 200 μl of PBS. Behavioral testing was performed 120 min after administration. Data are presented as box plots showing: interquartile range (the width of the box), minimum and maximum values (whiskers), and median (horizontal line). Each experimental group consisted of 12 animals. The statistical significance was evaluated using Mann–Whitney test

Repeated treatment with *L.* *helveticus* R0052 did not produce any significant changes in both the percentage of open arm entries (U = 63 p = 0.630; Fig. 5A) and the percentage of time spent in open arms (U = 70 p = 0.932, Fig. 5B) measured in the elevated plus maze test. Similarly, *L.* *helveticus* R0052 administration failed to affect the number of entries (U = 54, p = 0.475; Fig. 5C) and time spent in the light compartment (U = 70, p = 0.932; Fig. 5D) in the light/dark transition test. There were also no statistically significant differences in the latency to enter the dark compartment (U = 56, p = 0.369; Fig. 5E), though *L.* *helveticus* R0052 treatment caused ~ 65% increase in this parameter.

### Effect *L.* *helveticus* R0052 administration on locomotor activity, neuromuscular strength, and motor coordination

**Fig. 6:**
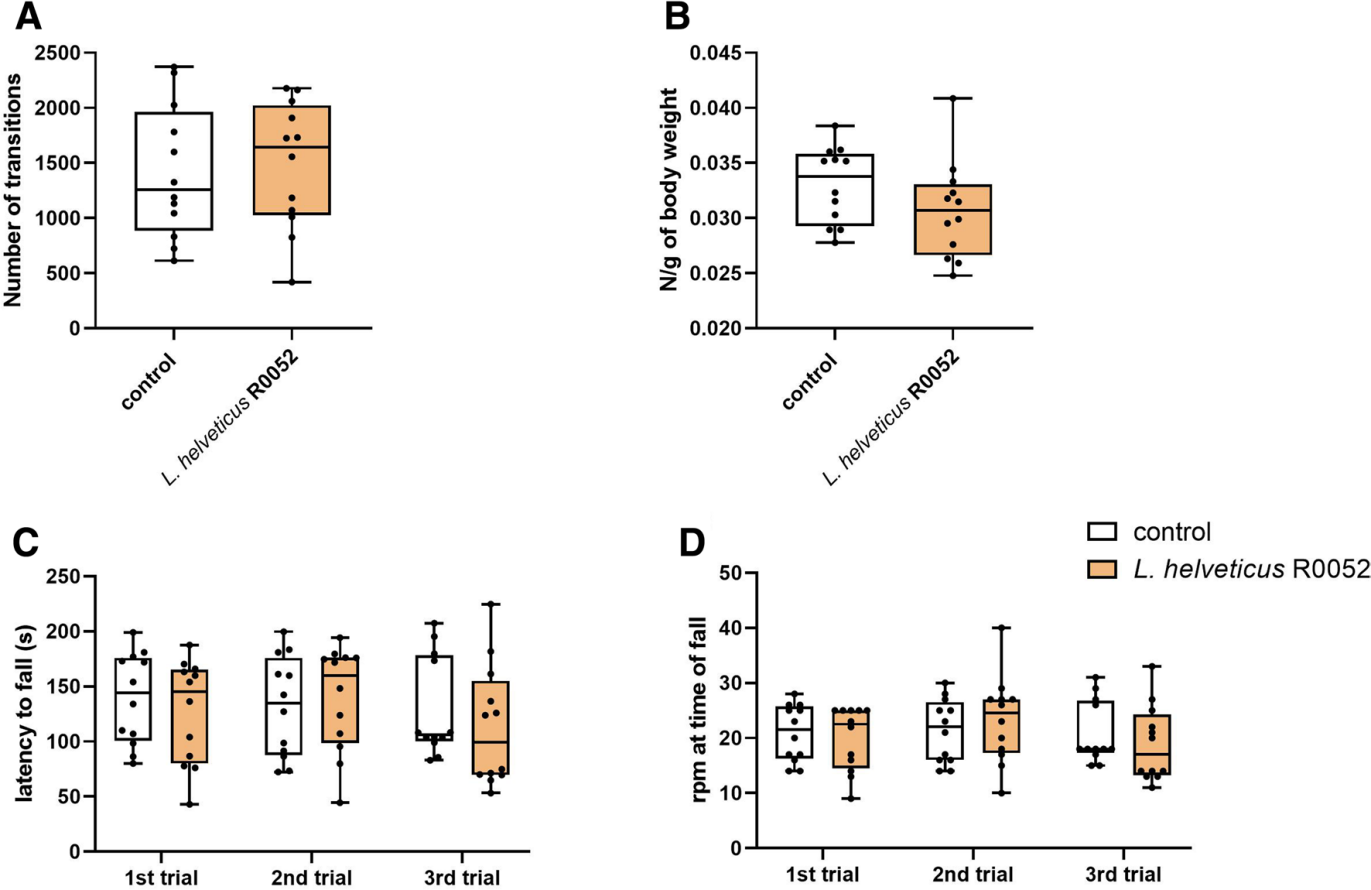
Effect of *L.* *helveticus* R0052 administration on locomotor activity (A), neuromuscular strength (B), and motor coordination (C and D). Mice were administered orally with 2 × 10^9^ cfu of *L.* *helveticus* R0052 for 27 days. Control animals received 200 μl of PBS. Behavioral testing was performed 120 min after administration. Data are presented as box plots showing: interquartile range (the width of the box), minimum and maximum values (whiskers), and median (horizontal line). Each experimental group consisted of 12 animals. The statistical significance was evaluated using Mann–Whitney test

No significant changes in spontaneous locomotor activity (U = 68, p = 0.843, Fig. 6A) and neuromuscular strength (U = 44, p = 0.110; Fig. 6B) in response to treatment with *L.* *helveticus* R0052 were reported in this study. Administration of *L.* *helveticus* R0052 did not also affect motor coordination in mice, as there were no changes in the latency to fall (first trial: U = 57, p = 0.410; second trial: U = 65, p = 0.713; third trial: U = 54, p = 0.319; Fig. 6C) and the rpm at time of fall (first trial: U = 60, p = 0.500; second trial: U = 60.5, p = 0.522; third trial: U = 53.5, p = 0.296; Fig. 6D) in the accelerating rotarod test.

### Effect *L.* *helveticus* R0052 administration on the cecal levels of SCFAs and lactic acid

**Fig. 7:**
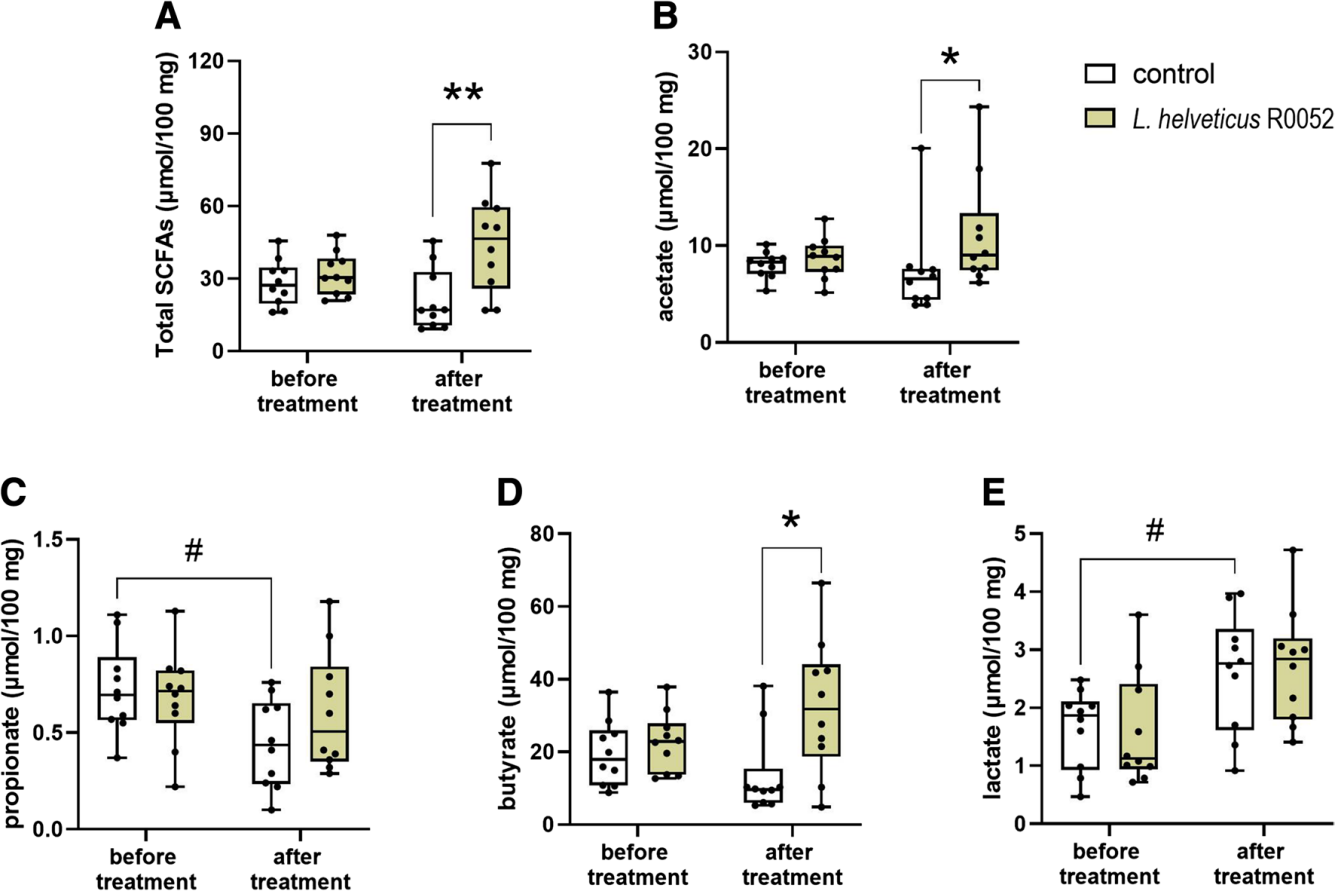
Effect of *L.* *helveticus* R0052 administration on the concentrations of total SCFAs (A), acetate (B), propionate (C), butyrate (D), and lactate (E). Mice were administered orally with 2 × 10^9^ cfu of *L.* *helveticus* R0052 for 28 days. Control animals received 200 μl of PBS. Fecal samples were collected before and after treatment. Data are presented as box plots showing: interquartile range (the width of the box), minimum and maximum values (whiskers), and median (horizontal line). Each experimental group consisted of 10 animals. The differences between control and *L.* *helveticus* R0052-treated group were evaluated using Mann–Whitney test, while the Wilcoxon matched-pairs signed rank test was used for before-after treatment comparison in the same mice. *p < 0.05, **p < 0.01 vs. control group; ^#^p < 0.05 vs. before treatment group

There were no statistically significant differences in concentrations of total SCFAs, acetate, propionate, butyrate, and lactate in fecal samples from mice assigned to the control group and *L.* *helveticus* R0052*-*treated group at the beginning of the experiment (i.e., before treatment). Administration of *L.* *helveticus* R0052 for 28 days caused a significant increase of total SCFAs (U = 16, p = 0.009; Fig. 7A), acetate (U = 19, p = 0.019; Fig. 7B), and butyrate (U = 20.5, p = 0.025; Fig. 7D) concentrations as compared to the control group. Propionate (U = 36, p = 0.306; Fig. 7C) and lactate (U = 49, p = 0.971; Fig. 7E) concentrations were not significantly affected in comparison to control animals.

Pairwise comparisons before and after treatment showed a statistically significant increase of lactate concentrations and a decrease of propionate concentration in fecal samples from PBS-treated mice (p = 0.020 and p = 0.047, respectively). No changes in the *L.* *helveticus* R0052*-*treated group were reported.

## Discussion

Three acute seizure tests were used to evaluate the effect of 28-day supplementation with a probiotic bacteria *L.* *helveticus* R0052 on the thresholds for different types of seizures in mice. First, we assessed its influence on the threshold for the maximal electroshock-induced seizures that mimic generalized clonic-tonic seizures in humans. We found that administration of *L.* *helveticus* R0052 did not affect the threshold for tonic extension of the hindlimbs in this test. However, *L.* *helveticus* R0052 significantly affected seizure susceptibility in the second model of electrically-induced seizures, i.e., in the 6 Hz test. In this test, low frequency and long-duration stimulation results in ‘psychomotor’ or ‘psychic’ seizures that, in contrast to maximal electroshock-induced seizures, are characterized by minimal clonic phase accompanied by automatized behaviors. The 6 Hz-induced psychomotor seizures are considered a model of human partial seizures (Barton et al. 2001). Interestingly, *L.* *helveticus* R0052 caused a marked (by 60%) increase of the CC_50_ value for psychomotor seizures. Next, we examined its effect in the timed *iv*PTZ seizure test which models myoclonic and generalized tonic–clonic seizures and is one of the most sensitive methods for evaluating seizure thresholds in rodents. Supplementation with *L.* *helveticus* R0052 slightly increased the threshold for the *iv*PTZ-induced first myoclonic twitch and generalized clonic seizure but it did not delay further progression of seizures as no changes in the threshold for tonic forelimb extension were observed.

There are several hypothetical mechanisms by which *L.* *helveticus* R0052 could increase seizure thresholds; these may include increased GABA level, altered GABAergic neurotransmission, reduced neuroinflammation, and ameliorated oxidative stress. Studies show that probiotics may produce beneficial effects in seizure/epilepsy by increasing GABA concentrations (Iannone et al. 2022). In the study by Bagheri et al. (2019), probiotic treatment considerably raised GABA concentration in the brain of the PTZ-kindled rats. Elevated serum GABA concentration following probiotic supplementation was also reported in human patients with drug-resistant epilepsy (Gómez-Eguílaz et al. 2018). Moreover, probiotics supplementation can alter GABA_A_ receptor expression in the brain (Bravo et al. 2011), which may also contribute to the antiseizure action. Unfortunately, we did not perform any additional assays to evaluate the effect of *L.* *helveticus* R0052 on GABA concentrations and/or GABA_A_ receptor expression to confirm its possible effects on GABA-mediated inhibitory neurotransmission. It should be however noticed that *L.* *helveticus* R0052 raised seizure thresholds in the *iv*PTZ test and seizures evoked by administration of PTZ are particularly sensitive to compounds that increase GABAergic transmission because the proconvulsant activity of PTZ results from its ability to block the chloride ion channel of the GABA_A_ receptor complex. This suggests possible influence of *L.* *helveticus* R0052 on inhibitory GABAergic neurotransmission.

Next, probiotics may induce antiseizure effects via anti-inflammatory and antioxidant mechanisms. For example, Wang et al. (2022) found that probiotic supplementation reduced spontaneous seizure in the kainic acid-induced epilepsy model and the effect was accompanied with decreased level of inflammatory cytokines (IL-1 β, IL-6, and TNF-α), lipid peroxidation, DNA damage, and increased total antioxidant ability in the hippocampus. Decreased brain levels of IL-6, TNF-α, and nitric oxide following probiotic treatment were also reported in WAG/Rij rats (Aygun et al. 2022b) and in rats subjected to the PTZ seizure test (Aygun et al. 2022a). In a study by Wang et al. (2019b), *L.* *helveticus* R0052 exhibited anti-inflammatory properties by downregulating TLR2, TLR4, TLR5, NF-κB, and TNF-α transcription in liver, decreasing proinflammatory cytokines (IL-2, IL-6, IL-12, IL-17, TNF-α, RANTES, and MIP-3α) in serum, and enhancing the intestinal barrier of d-galactosamine-treated rats. The anti-inflammatory properties of *L.* *helveticus* R0052, in combination with *B.* *longum* R0175, were also demonstrated in other studies (De Oliveira et al. 2023; Mohammadi et al. 2019; Partrick et al. 2021).

Importantly, we found that *L.* *helveticus* R0052 increases fecal SCFAs content. Acetate, propionate, and butyrate are the most abundant SCFAs produced in the colon during microbial fermentation of dietary fibers. SCFAs are highly implicated in inflammatory responses, maintenance of intestinal and blood–brain barrier integrity, and in neuromodulation. Evidence shows that the gut microbiota-derived SCFAs may affect seizure susceptibility. In the study by Ohland et al. (2013), *L.* *helveticus* R0052 did not significantly affect SCFAs concentrations in either normal diet or Western-diet fed mice suggesting that this bacteria strain does not exert probiotic effects by alterations in carbohydrate metabolism. By contrast, we found that a 28-day supplementation with* L. helveticus* R0052 increases concentrations of total SCFAs, acetate, and butyrate as compared to the control group, which indicates that the elevation of seizure thresholds in the 6 Hz and *iv*PTZ tests could be, at least in part, associated with increased SCFAs concentrations.

We did not observe any changes in SCFAs and lactate concentrations in the same animals before and after *L.* *helveticus* R0052 treatment. In PBS-treated mice, a decrease of propionate concentration and an increase of lactate concentration were reported. It should be however noticed that numerous factors (including handling and stress caused by oral gavage) may affect the gut microbiota profile and thereby production of SCFAs (and other metabolites) in rodents, which introduces a potential confound and may lead to misinterpretation of the data (Allen-Blevins et al. 2017). For this reason, comparing the levels of SCFAs in the same animals before and after treatment may not be a reasonable approach.

Since anxiety is one of the most common psychiatric comorbidities in epileptic patients, we also evaluated the effect of *L.* *helveticus* R0052 on the anxiety-like behavior in mice. It was shown that *L.* *helveticus* R0052 in combination with *B. longum* R0175 may produce psychotropic-like effects both in animals and humans. This probiotic mixture alleviated stress responses in rats (Ait-Belgnaoui et al. 2014, 2018), reduced symptoms of the post-myocardial infarction depression in rats (Arseneault-Bréard et al. 2012), decreased anxiety-like behavior in the conditioned defensive burying test in rats (Messaoudi et al. 2011), reduced psychological distress in healthy human volunteers (Messaoudi et al. 2011), and improved depression symptoms in patients with major depressive disorder (Kazemi et al. 2019). Also, *L.* *helveticus* R0052 administered alone reduced the anxiety-like behavior assessed in the Barnes maze test in mice fed Western-type diet (Ohland et al. 2013). Our results do not confirm the anxiolytic-like properties of *L.* *helveticus* R0052 as none of the anxiety-related parameters measured in the elevated plus maze and the light/dark transition paradigm were significantly improved. It is noteworthy that the effect of *L.* *helveticus* R0052 on mouse behavior in the study by Ohland et al. (2013) was in fact diet- and genotype-dependent. A 21-day supplementation with *L.* *helveticus* R0052 prevented the increased anxiety-like behavior induced by a Western-style diet in both the wild-type mice and the interleukin-10 deficient (IL-10^−/−^) 129/SvEv mice with immune system dysregulation. By contrast, ingestion of *L.* *helveticus* R0052 had anxiogenic-like effect in the wild-type mice fed standard diet and no effect on anxiety-related behavior in the standard diet-fed IL-10^−/−^ mice (Ohland et al. 2013). Moreover, evidence shows that the effects of probiotics may differ across species or animal strains. For example, a well-known psychobiotic *Lactobacillus rhamnosus* JB-1 differentially affected the anxiety-like behavior in Balb/c and C57BL/6 mice (Bharwani et al. 2017). Moreover, the aforementioned probiotic mixture containing *L.* *helveticus* R0052 and *B. longum* R0175 increased anxiety-like behavior and changes following social defeat stress in Syrian hamsters (Partrick et al. 2021), which is in contrast to the previously reported anxiolytic-like effects.

To further characterize the effects of *L.* *helveticus* R0052, we assessed its influence on motor performance in the accelerating rotarod test, spontaneous locomotor activity, and neuromuscular strength. No significant changes in motor coordination, locomotor activity or grip strength were reported.

The gut microbiota is also implicated in the metabolism of many drugs, thereby alteration of gut microbiota can affect bioavailability and efficacy of some drugs. On the other hand, medicines may change the gut microbiota composition (Iannone et al. 2020). Several antiseizure drugs were shown to affect the growth of intestinal bacteria species in vitro (Esiobu and Hoosein 2003; Ilhan et al. 2022) and alter the gut microbiota composition in vivo (Cussotto et al. 2019; Watanangura et al. 2022). It was also demonstrated that intestinal microbiota may be involved in the metabolism of clonazepam (Zimmermann et al. 2019) and zonisamide (Kitamura et al. 1997). Moreover, the antiseizure activity of valproate was reduced in mice with dextran sulfate sodium-induced colitis (De Caro et al. 2019b) suggesting that the gut dysbiosis associated with colitis (Munyaka et al. 2016) may affect therapeutic efficacy of antiseizure drugs. Unfortunately, studies about the effects of gut microbiota manipulation by probiotics/prebiotics on the activity and/or metabolism of antiseizure drugs are missing. In this study, we showed that a 28-day supplementation with *L.* *helveticus* R0052 did not affect the antiseizure activity of valproate in the *sc*PTZ test in mice. Interestingly, we found that *L.* *helveticus* R0052 treatment significantly increased valproate concentration in serum, but not in the brain. The increased oral bioavailability of valproate after chronic administration of *L.* *helveticus* R0052 may be explained by the fact that this drug, besides beta oxidation in the mitochondria and cytochrome P450-mediated oxidation, is in about 50% metabolized by glucuronidation. In vitro studies of human liver microsomes and purified recombinant proteins have reported glucuronidation of valproic acid by UGT1A3, UGT1A4, UGT1A6, UGT1A8, UGT1A9, UGT1A10, and UGT2B7 (Argikar and Remmel 2009; Ethell et al. 2003). Some of these enzymes, namely UGT1A1, UGT1A8, UGT1A9, and UGT1A10 are present in the human intestine. Therefore, it is suggested that intestinal glucuronidation catalyzed by UGTs, particularly UGT1A8 and UGT1A10, may play important roles in the first-pass metabolism of drugs, especially carboxylic acid drugs. The tissue distribution of the Ugt1a family isoforms in mice and rats is similar to that observed in humans. In addition, most of the substrates of human UGTs are glucuronidated by mouse Ugt family enzymes (Fujiwara et al. 2018). In a study in rats, one of the most widely used probiotics, *L. rhamnosus* R0011 promoted the bioavailability of glycyrrhizinic and glycyrrhetinic acid, especially under liver fibrosis state. In the same study, it was shown that fecal glucuronidase activity was significantly increased in rats treated with *L. rhamnosus* R0011 at a dose of 1 ml (1 × 10^9^ CFU/ml) once daily for 1 week (Li et al. 2022). Thus, it can be assumed that in the present study supplementation with *L.* *helveticus* R0052 may have directly participated in valproic acid biotransformation, e.g. via an increased activity of intestinal β-glucuronidase leading to the slightly enhanced bioavailability after oral dosing.

An important question in terms of probiotics use is whether multi-strain mixtures are more effective than single-strain preparations. An analysis performed by McFarland (2021) showed that single strain probiotics are generally equivalent to mixtures and it appears that the choice of probiotic should be based on the evidence of its efficacy in the specific condition rather than on the number of strains in the preparation. As mentioned above, several animal and human studies showed that probiotics supplementation may reduce the number of seizures and/or reduce seizure severity. In all of these studies, multi-strain probiotics were used. Here, we showed that a single-strain probiotic *L. helveticus* R0052 may also affect seizure susceptibility and produce potential beneficial effects by increasing SCFAs production. Interestingly, the meta-analysis demonstrated that *L. helveticus* R0052 as a single strain preparation significantly prevented antibiotic-associated diarrhea in humans, whereas the addition of another probiotic strain did not improve the efficacy (McFarland 2021). Nevertheless, it would be advisable to investigate in further studies the effect of a multi-strain probiotic containing of *L. helveticus* R0052 on seizure susceptibility and efficacy of antiseizure drugs.

In summary, results obtained in the present study provide further support for potential beneficial effects of probiotic supplementation in epilepsy management. We showed that single-strain probiotic supplementation may decrease seizure susceptibility in mice. A 28-day treatment with *L.* *helveticus* R0052 increased the threshold for psychomotor seizure. There was also a slight increase in the threshold for myoclonic and clonic seizure. The effect could be partially related with increased production of SCFAs that may alter seizure activity by modulating excitatory/inhibitory neurotransmission, oxidative stress, and neuroinflammation. (Kim et al. 2022). Importantly, we also found that supplementation with *L.* *helveticus* R0052 may affect bioavailability of sodium valproate.

## Data Availability

The data that support the findings of this study are available from the corresponding author upon reasonable request.
